# Origins of Context-Dependent Gene Repression by Capicua

**DOI:** 10.1371/journal.pgen.1004902

**Published:** 2015-01-08

**Authors:** Marta Forés, Leiore Ajuria, Núria Samper, Sergio Astigarraga, Claudia Nieva, Rona Grossman, Sergio González-Crespo, Ze'ev Paroush, Gerardo Jiménez

**Affiliations:** 1 Institut de Biologia Molecular de Barcelona-CSIC, Parc Científic de Barcelona, Barcelona, Spain; 2 Department of Developmental Biology and Cancer Research, IMRIC, The Hebrew University, Jerusalem, Israel; 3 Institució Catalana de Recerca i Estudis Avançats, Barcelona, Spain; Harvard Medical School, Howard Hughes Medical Institute, United States of America

## Abstract

Receptor Tyrosine Kinase (RTK) signaling pathways induce multiple biological responses, often by regulating the expression of downstream genes. The HMG-box protein Capicua (Cic) is a transcriptional repressor that is downregulated in response to RTK signaling, thereby enabling RTK-dependent induction of Cic targets. In both *Drosophila* and mammals, Cic is expressed as two isoforms, long (Cic-L) and short (Cic-S), whose functional significance and mechanism of action are not well understood. Here we show that *Drosophila* Cic relies on the Groucho (Gro) corepressor during its function in the early embryo, but not during other stages of development. This Gro-dependent mechanism requires a short peptide motif, unique to Cic-S and designated N2, which is distinct from other previously defined Gro-interacting motifs and functions as an autonomous, transferable repressor element. Unexpectedly, our data indicate that the N2 motif is an evolutionary innovation that originated within dipteran insects, as the Cic-S isoform evolved from an ancestral Cic-L-type form. Accordingly, the Cic-L isoform lacking the N2 motif is completely inactive in early *Drosophila* embryos, indicating that the N2 motif endowed Cic-S with a novel Gro-dependent activity that is obligatory at this stage. We suggest that Cic-S and Gro coregulatory functions have facilitated the evolution of the complex transcriptional network regulated by Torso RTK signaling in modern fly embryos. Notably, our results also imply that mammalian Cic proteins are unlikely to act via Gro and that their Cic-S isoform must have evolved independently of fly Cic-S. Thus, Cic proteins employ distinct repressor mechanisms that are associated with discrete structural changes in the evolutionary history of this protein family.

## Author Summary

Understanding the evolution of developmental regulatory mechanisms is a central challenge of biology. Here we uncover a newly evolved mechanism of transcriptional repression by Capicua (Cic), a conserved sensor of Receptor Tyrosine Kinase (RTK) signaling. In *Drosophila*, Cic patterns the central regions of the embryo by repressing genes induced by Torso RTK signaling at the poles. We show that Cic performs this function by recruiting the Groucho (Gro) corepressor and that this mechanism is an evolutionary innovation of dipteran insects. Indeed, we find that recruitment of Gro depends on a short motif of Cic (N2) specific to dipterans. Strikingly, moreover, the form of Cic that existed before the origin of dipterans is completely inactive in fly embryos, whereas the equivalent form carrying N2 displays significant function. This suggests that evolution of the N2 motif caused a fundamental change in Cic repressor activity, which we propose has enabled the complex roles of Cic, Gro and Torso signaling in fly embryonic patterning. In contrast, Cic functions independently of Gro in other *Drosophila* tissues and probably also in mammals, where Cic lacks the N2 sequence. Thus, our results illustrate the structural and evolutionary origins of essential functional variations within a highly conserved family of developmental regulators.

## Introduction

Receptor Tyrosine Kinase (RTK) signaling pathways regulate tissue development and morphogenesis in all metazoans [1]. RTKs often signal through the conserved Ras-Raf-MAPK cascade, leading to phosphorylation of nuclear transcription factors which then elicit changes in target gene expression. The HMG-box protein Capicua (Cic) has recently emerged as a general nuclear sensor of RTK signaling pathways [2]. Originally discovered downstream of the Torso RTK in *Drosophila* embryogenesis, Cic has been subsequently shown to function downstream of other RTKs at multiple stages of fly development [3]–[11]. In all cases, Cic represses transcription of RTK-responsive genes in unstimulated cells, whereas activation of RTK signaling results in phosphorylation and downregulation of Cic and this causes derepression of its target genes [7], [10], [12], [13].

Cic is highly conserved from cnidarians to vertebrates and is implicated in several human pathologies such as spinocerebellar ataxia type 1 (SCA1) and oligodendroglioma (OD) [14]–[17]; reviewed in [2]. Indeed, Cic proteins from *Drosophila* and mammals share many functional and structural properties: they repress transcription by binding to related DNA sites in target genes, appear to be similarly downregulated by RTKs and are expressed as two main isoforms, short (Cic-S) and long (Cic-L), which differ in their N-terminal regions [7], [9], [10], [14], [15], [17]–[19]. However, despite these similarities, it is currently unclear whether all Cic family proteins employ a common mechanism of repression. Studies in mouse and human cells have shown that Cic associates with Ataxin1 (Atxn1), a co-repressor involved in SCA1 [14], [15], [17], [20], [21]. On the other hand, previous studies in *Drosophila* have suggested that Cic functions together with Groucho (Gro) [3], [10], a WD-repeat co-repressor that associates with multiple repressors, including Hairy/Hes, Nkx, Lef/Tcf and Runx family proteins (reviewed in [22], [23]). However, the functional links between Cic and Gro remain unclear, since no molecular interaction between these proteins has been validated in vivo [2], [24].

Here, we investigate the mechanism of *Drosophila* Cic repression and its relationship with Gro. We find that Cic functions via Gro in the early embryo but not at other developmental stages. The Gro-assisted mechanism depends on a previously unrecognized motif of Cic (N2), which is essential for recruitment of Gro in vivo. Remarkably, the N2 motif is highly conserved among Cic orthologues in flies and mosquitoes, but is absent in all other species, suggesting that it originated in ancestral dipterans. Furthermore, the N2 domain appears to be a structural innovation associated with the emergence of fly Cic-S isoforms from a pre-existing Cic-L-like isoform. This implies that mammalian Cic proteins, which lack the N2 motif, probably function independently of Gro, and that their Cic-S isoforms must have evolved independently of fly Cic-S. Thus, Cic proteins exhibit context-dependent repressor activities that are partly associated with key structural changes that have occurred during the evolution of this protein family.

## Results

### Context-dependent activities of Cic in *Drosophila* development

**Figure 1:**
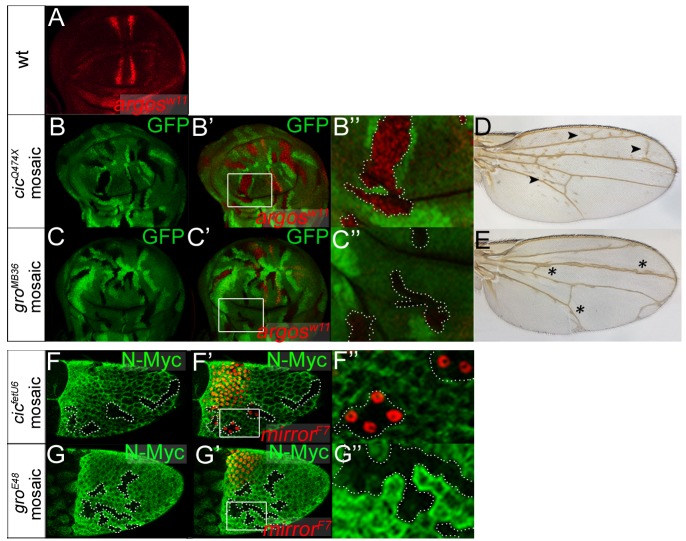
Cic functions independently of Gro in the ovary and in the wing. (A) Expression of *argos* in a third instar wing imaginal disc as revealed by LacZ (β-galactosidase) immunostaining using the *argos^W11^–lacZ* enhancer trap. Expression is detected in presumptive vein stripes where EGFR signaling is active, and is absent in intervein regions where Cic represses *argos*. (B-C″) Mosaic wing imaginal discs carrying *cic^Q474X^* (B-B″) and *gro^MB36^* (C-C″) mutant clones marked by absence of GFP (green, outlined in B″ and C″). B′ and C′ show merged images of GFP signals and *argos^W11^–lacZ* expression (red); B″ and C″ show close-ups of boxed areas in panels B′ and C′. Note that loss of Cic function leads to full derepression of *argos^W11^–lacZ* in the mutant clones, whereas the loss of Gro causes derepression of *argos^W11^–lacZ* only in close proximity to its normal stripes of expression. This localized effect of Gro probably reflects its role together with Enhancer-of-split/Hes repressors in refining *argos* expression [45]. (D and E) Mosaic adult wings carrying *cic^Q474X^* (D) and *gro^MB36^* (E) mutant clones induced in third instar larvae as above. Consistent with the effects on *argos^W11^–lacZ* expression, the phenotypes of *cic^Q474X^* and *gro^MB36^* mosaic wings are clearly different: *cic* mosaic wings show patches of ectopic vein material throughout the wing blade (arrowheads), whereas *gro* mosaic wings display localized thickening of veins (asterisks). This indicates that Cic repression in the developing wing does not rely on Gro. (F-G″) Stage-10 mosaic egg chambers carrying *cic^fetU6^* (F-F″) and *gro^E48^* (G-G″) mutant clones marked by absence of N-Myc immunofluorescence (green, outlined in F″ and G″). F′ and G′ show merged images of N-Myc signals and *mirror* expression visualized using the *mirror^F7^–lacZ* enhancer trap and anti-LacZ staining (red). F″ and G″ show close-ups of boxed areas in panels F′ and G′. *mirror* is a key regulator of dorsoventral axis formation that is activated by EGFR signaling in dorsal-anterior follicle cells, and repressed by Cic in ventral follicle cells. *cic* loss-of-function clones in ventral regions cause derepression of *mirror^F7^–lacZ*, although only in the anterior half of the follicular epithelium [4], [6]. In contrast, *gro* mutant clones do not show *mirror^F7^–lacZ* derepression, suggesting that Cic also acts independently of Gro in this context.

Cic and Gro are both essential for repression of two terminal gap genes, *tailless* (*tll*) and *huckebein* (*hkb*), in central regions of the blastoderm embryo; this repression is normally relieved by Torso RTK signaling at the embryonic termini, thereby enabling localized induction of *tll* and *hkb* by broadly distributed activators [3], [4], [25]. These shared requirements of Cic and Gro in the terminal system have led to the idea that both proteins act in a common repressor complex (see refs. [2], [24]). However, we have assayed the requirement of Gro for Cic repressor functions in other developmental contexts and found that Gro is dispensable for such functions (Fig. 1). Specifically, we examined two systems -the developing wing and the ovarian follicular epithelium- where Cic represses specific target genes such as *argos* and *mirror*, respectively, under the control of the EGFR pathway [4]–[6], [10]–[12]. In these experiments, we compared the effects caused by the loss of Cic or Gro function using mosaic analyses. Unexpectedly, we found that loss of Gro function does not impair Cic repression in any of those systems, indicating that Cic represses *argos* and *mirror* independently of Gro (Fig. 1).

**Figure 2:**
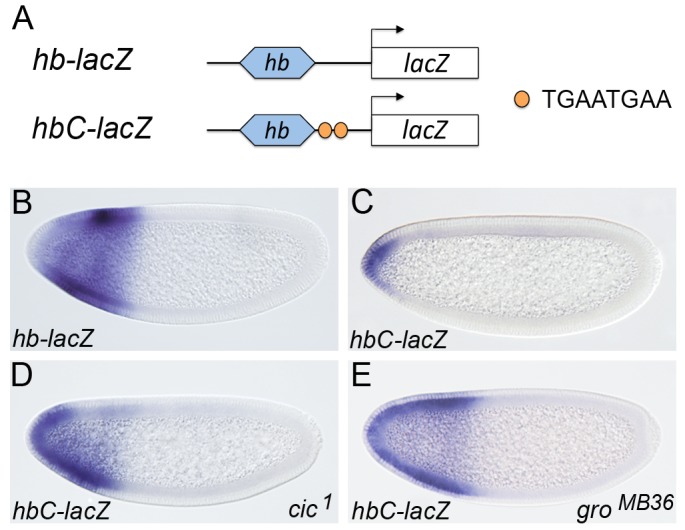
Cic binding sites are sufficient for recruitment of Gro in vivo. (A) Diagram of *lacZ* transgenes under the control of a minimal *hb* enhancer and canonical Cic binding sites (TGAATGAA). (B-E) mRNA expression patterns of *hb-lacZ* and *hbC-lacZ* in otherwise wild-type (B, C), *cic^1^* (D) or *gro^MB36^* (E) mutant embryos; note the strong derepression of *hbC-lacZ* in both mutant backgrounds. In this and subsequent figures, anterior is to the left and dorsal is up.

In light of these results, we have re-evaluated the functional links between Cic and Gro in the early embryo. First, we asked if Cic-mediated repression of a synthetic reporter gene relies on Gro activity in the early embryo. To this end, we used a transgenic construct containing a minimal *hunchback* (*hb*) enhancer linked to a pair of individual Cic binding sites (*hbC*; ref. [10]) (Fig. 2A). The intact *hb* enhancer drives broad expression in the anterior third of the embryo (Fig. 2B), whereas *hbC* is repressed by Cic and drives expression only in the anterior pole of the embryo, where Cic is downregulated by Torso RTK signaling (Fig. 2C, D). As shown in Fig. 2E, we find clear derepression of *hbC* activity in embryos lacking Gro function, implying that Cic represses *hbC* via Gro in this assay. These results support the idea that Cic indeed acts through Gro in early embryonic patterning.

### N2, a new motif of Cic that is essential for repression

**Figure 3:**
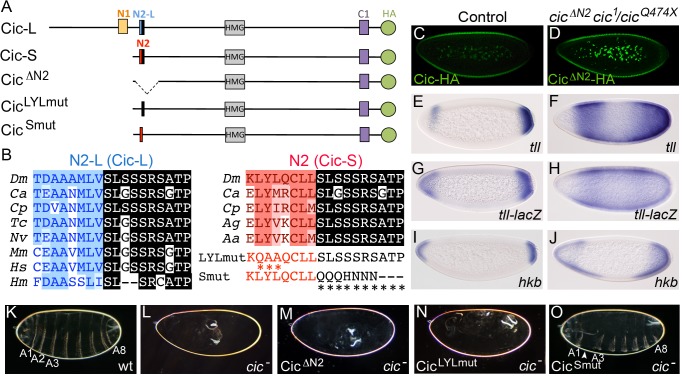
The N2 motif is essential for Cic embryonic function. (A) Diagram of *Drosophila* Cic-L and Cic-S isoforms and three derivatives carrying mutations in the N2 motif. Cic-L and Cic-S are generated via use of alternative promoters and splicing sites, which produce different N-terminal domains. At the site of alternative splicing, Cic-L and Cic-S contain two different conserved motifs, N2-L and N2, which include different N-terminal sequences (shown in blue and red, respectively) and a common C-terminal peptide (highlighted in black). Other conserved domains (including the N1 and C1 domains of unknown function) are also indicated. The proteins are shown with an HA tag (green) to allow their visualization in transgenic embryos (see also below). (B) Alignment of Cic N2-L and N2 sequences from different species. *Dm*, *Drosophila melanogaster*; *Ca*, *Clogmia albipunctata*; *Cp*, *Culex pipiens*; *Tc*, *Tribolium castaneum*; *Nv*, *Nasonia vitripennis*; *Mm*, *Mus musculus*; *Hs*, *Homo sapiens*; *Hm*, *Hydra magnipapillata; Ag*, *Anopheles gambiae*; *Aa*, *Aedes aegypti*. Two different mutations of the N2 peptide (LYLmut and Smut) are also shown below the N2 alignment. (C and D) Expression of Cic and Cic^ΔN2^ proteins tagged with the HA epitope in embryos stained with anti-HA antibody; note that both proteins appear downregulated at the embryo poles. (E-M) mRNA expression patterns of *tll*, *tll-lacZ* and *hkb* in wt (E, G, I) and *cic* mutant (*cic^1^/cic^Q474X^*) embryos expressing Cic^ΔN2^ (F, H, J). Cuticle phenotypes of the same genetic backgrounds are shown in K and M, respectively; panel L shows a control *cic^1^/cic^Q474X^* mutant cuticle. (N and O) Cuticle phenotypes of *cic^1^/cic^Q474X^* mutant embryos expressing the Cic^LYLmut^ (N) and Cic^Smut^ (O) derivatives; only Cic^Smut^ rescues the *cic* phenotype, except for mild segmental defects (arrowhead). A1-A8, abdominal segments 1–8.

Cic does not contain either of the two previously defined Gro-binding motifs present in known Gro-dependent repressors, the WRPW- and eh1-like peptides [22], and we have not detected direct interactions between functionally important regions of Cic and Gro [10]. Therefore, we asked what sequences of Cic mediate its Gro-dependent repressor activity. Assuming that those sequences could be evolutionarily conserved, we noted a novel conserved motif present at the N-terminus of the Cic-S isoform (GenBank protein AAF55751), which we designate N2 (Fig. 3A, B). This motif is encoded in two adjacent exons: a 5′ exon specific of the *cic-S* transcript and a 3′ exon shared by both *cic-S* and *cic-L* transcripts (see also below). The sequence encoded by the *cic-S*-specific exon (LYLQCLL) is conserved in dipteran species (Fig. 3A, B, highlighted in red), whereas the peptide common to Cic-S and Cic-L isoforms (SLSSSRSATP) is conserved from hydra to humans (Fig. 3A, B, highlighted in black). To assess the functional significance of N2, we assayed the activity of a Cic-S derivative lacking this motif (Cic^ΔN2^). We find that Cic^ΔN2^ is expressed at normal levels in transgenic embryos but is unable to repress *tll*, a *tll* reporter or *hkb* (Fig. 3C-J). Accordingly, Cic^ΔN2^ does not provide any rescue of the *cic* embryonic mutant phenotype (Fig. 3K-M), indicating that N2 is critical for Cic function in the early embryo.

We also tested two mutations affecting each of the sub-elements of N2. Surprisingly, disruption of the Cic-S-specific element caused a complete loss of Cic-S function, whereas mutation of the second, highly conserved sequence had a minor effect on protein activity (Fig. 3N, O). Thus, only the dipteran-specific portion of N2 is essential for Cic embryonic function.

### N2 is a Gro-dependent repressor element

**Figure 4:**
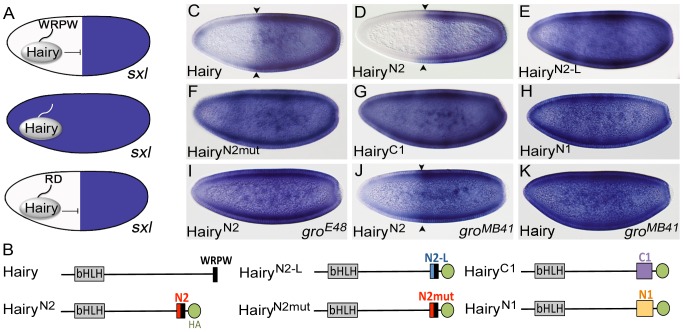
N2 is a Gro-dependent repressor motif. (A) Schematic representation of the *Sxl* repression assay. In this assay, expression of Hairy under the control of the *hb* promoter in the anterior region of the embryo leads to repression of *Sxl* transcription (blue) in females (top). Repression depends on the WRPW motif of Hairy (middle), but replacement of this motif with autonomous repressor domains (RD) restores repression function (bottom). (B) Diagram of Hairy and Hairy fusion constructs tested in the *Sxl* assay; all fusions carry a C-terminal HA tag (see Materials and Methods). (C-K) Effects of Hairy constructs on *Sxl* expression in otherwise wild-type or *gro* mutant embryos; all images correspond to *Sxl* expression in female embryos. Arrowheads indicate borders of transcriptional repression. Note that Hairy^N2^ does not cause complete repression of *Sxl* in g*ro^MB41^* embryos (J); this may reflect a loss of function, in this genetic background, for endogenous Hairy-related factors such as Deadpan that normally contribute to *Sxl* repression [46].

Based on the above results, we hypothesized that N2 could be involved in recruiting Gro to Cic target genes. In fact, the critical N2 sequence shares some similarity with the consensus eh1 motif (FxIxxIL) that binds directly to Gro, although it lacks the characteristic phenylalanine residue at position 1. We therefore tested if N2 functions as an autonomous, transferable Gro-dependent motif capable of imposing repressor activity on a heterologous DNA-binding domain. For this, we adopted the *Sex-lethal* (*Sxl*) repression assay, an in vivo strategy for analyzing the activity of known or potential repressor domains [26], [27]. In this assay, a domain under analysis is used to replace the Gro-binding WRPW motif of the Hairy repressor and tested for its ability to repress *Sxl* expression in the embryo (Fig. 4A). Using this approach, we found that a Hairy chimera carrying the N2 motif instead of the WRPW peptide (Hairy^N2^) represses *Sxl* as efficiently as intact Hairy (Fig. 4B-D). In contrast, four control Hairy chimeras carrying a mutant version of N2 or other conserved motifs from Cic, did not (Fig. 4B, E-H; see also S1 Fig.). Moreover, repression by the Hairy^N2^ chimera depends on Gro, as it is lost in *gro^E48^* mutant embryos that lack Gro activity (Fig. 4I). This indicates that N2 is a discrete, Gro-dependent repressor motif.

We also analyzed the activity of Hairy^N2^ in the presence of a Gro mutant protein, Gro^MB41^, which can not bind to WRPW or eh1 motifs but retains normal function in the terminal system (potentially acting together with Cic) [28]. The Gro^MB41^ mutant carries an amino acid substitution (R483H) affecting the central pore of the Gro β propeller domain, thereby preventing binding of WRPW or eh1 motifs across this pore [28]. We found that Hairy^N2^ displays significant repressor activity in g*ro^MB41^* embryos, whereas native Hairy is completely inactive in this background (Fig. 4J, K). Thus, Gro^MB41^ is functional both in repressing terminal gap genes and in mediating repression by Hairy^N2^, suggesting that it is recruited in each of these systems through similar interactions that involve the N2 motif.

**Figure 5:**
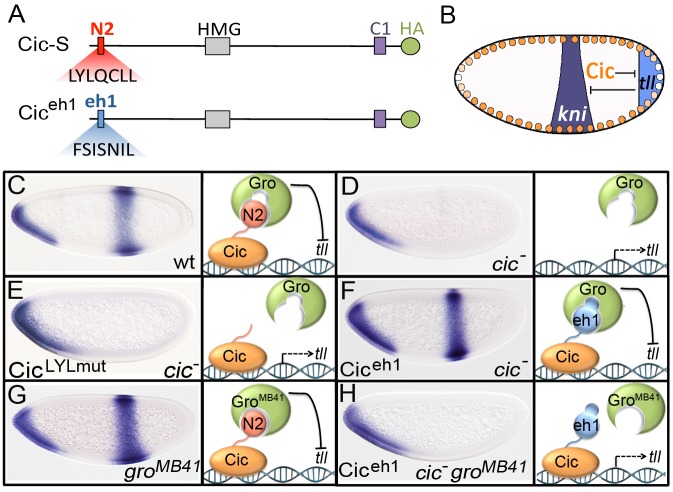
The N2 motif of Cic recruits Gro to the terminal patterning system. (A) Diagram of Cic and Cic^eh1^ proteins; Cic^eh1^ carries the eh1 motif from *Drosophila* Engrailed instead of N2 and is tagged with an HA epitope at the C-terminus. (B) Schematic representation of cross-repressive interactions between Cic, *tll* and *kni* in the early blastoderm. (C-H) mRNA expression patterns of *kni* in wild-type (C), *cic* (D, E, F), *gro^MB41^* (G) and *cic gro^MB41^* (H) mutant backgrounds expressing the Cic^LYLmut^ (E) and Cic^eh1^ (F, H) products. A model diagram depicting the interactions of N2 and eh1 motifs with Gro proteins and the resulting repressor activities is shown next to each embryo; for simplicity, the interaction between N2 and Gro is modeled as being direct (see Discussion). The *cic* maternal mutant genotypes are *cic^1^* for panels D, F and H, and *cic^1^/cic^Q474X^* for panel E.

As an independent test of this idea, we analyzed a Cic derivative in which the N2 sequence was replaced by the eh1 motif (FSISNIL) from the Engrailed homeodomain protein (Cic^eh1^; Fig. 5A). If Gro is recruited to the terminal system through the N2 motif, replacing this motif by the eh1 element should render Gro^MB41^ non-functional in that system. For these experiments, we monitored the expression of the central gap gene *knirps* (*kni*) as a sensitive readout of Cic and Cic^eh1^ repressor activities (Fig. 5B). *kni* is a target of the Tll repressor. When Cic is active, it restricts *tll* expression to the posterior pole of the embryo, thereby permitting expression of *kni* in the presumptive abdomen (Fig. 5B, C). In contrast, loss of Cic function causes derepression of *tll* and corresponding loss of the central *kni* stripe (Fig. 5D, E). We find that Cic^eh1^ is an active repressor capable of rescuing *kni* expression in *cic* mutant embryos (Fig. 5F), indicating that the eh1 peptide can compensate for the loss of endogenous N2 in its normal setting. We then compared *kni* expression in g*ro^MB41^* embryos expressing either endogenous Cic or Cic^eh1^. As previously reported, *kni* expression is normal in the first case (ref. [28]; Fig. 5G), whereas there is clear loss of *kni* expression in the presence of Cic^eh1^ (Fig. 5H). Therefore, it is the presence of an intact N2 motif in Cic that enables Gro^MB41^ to be functional in the terminal system, supporting our conclusion that N2 links Cic and Gro in the *Drosophila* embryo.

### Origin of N2 and Cic-S in dipterans

**Figure 6:**
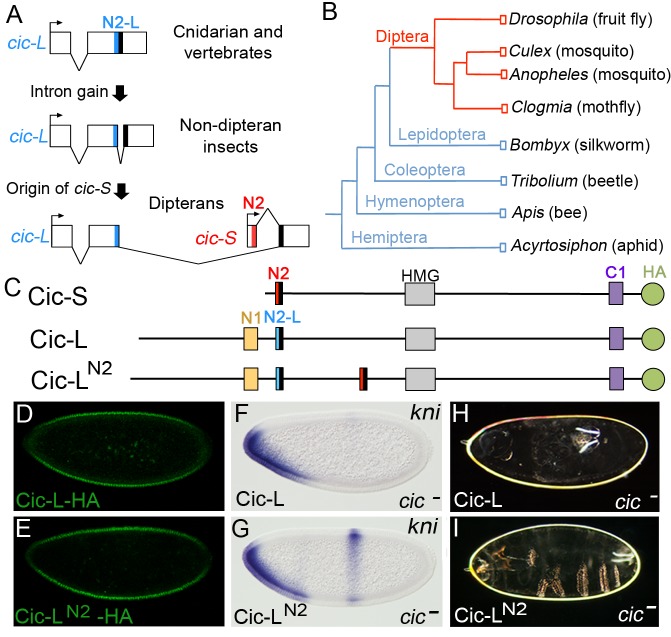
Recent origin of the N2 motif in dipterans. (A) Schematic representation of proposed steps giving rise to the Cic-S isoform and the N2 motif. The diagrams show the regions spanning the alternatively spliced exons of *cic-S* and *cic-L* transcripts in selected species, which are represented graphically and are not drawn to scale. The size of introns splitting the N2-L coding sequences in non-dipteran species are as follows: *Bombyx mori* (758 bp), *Tribolium castaneum* (50 bp), *Apis mellifera* (86 bp) and *Acyrthosiphon pisum* (129 bp) (see also main text). The conserved protein motifs encoded at relevant exon junctions are shaded in red, blue or black as in Fig. 3. (B) Insect phylogeny illustrating the presence of the Cic-S and N2 motifs in dipterans (red). (C) Diagram of the Cic-S, Cic-L and Cic-L^N2^ proteins; Cic-L^N2^ carries the N2 motif inserted within a poorly conserved sequence of Cic. Cic-L and Cic-L^N2^ were expressed with an HA tag at the C-terminus. (D and E) Expression of HA-tagged Cic-L (D) and Cic-L^N2^ (E) proteins in embryos stained with anti-HA antibody. (F and G) mRNA expression patterns of *kni* in *cic^1^/cic^Q474X^* mutant embryos expressing Cic-L (F) and Cic-L^N2^ (G); only Cic-L^N2^ leads to significant rescue of the central *kni* stripe. (H and I) Cuticle phenotypes of the same genetic backgrounds shown in F and G, respectively.

As indicated above, the key repressor element within the N2 motif is specific to the Cic-S isoform and is present only in dipterans. To get further insight into the evolution of this element, we examined the structure of the *cic* locus in different insect taxa, focusing on the region that spans the alternatively spliced exons of *Drosophila cic-S* and *cic-L* transcripts. We were able to perform these analyses given the high conservation of peptide sequences encoded by these alternative exon junctions (Fig. 3B). We found a similar *cic* genomic organization in *Drosophila* and four distant species of lower dipterans: *Clogmia albipunctata*, *Culex pipiens, Anopheles gambiae* and *Aedes aegypti* (Fig. 6A; S2 Fig.), implying that this organization was already present in an early common ancestor of dipterans. In contrast, a different structure, which lacks the first *cic-S* exon, is apparent across representative species of non-dipteran taxa, including *Bombyx mori* (Lepidoptera), *Tribolium castaneum* (Coleoptera), *Apis mellifera* (Hymenoptera), and *Acyrthosiphon pisum* (Hemiptera) (Fig. 6A, B). In this configuration, the two exons encoding the N2-L motif of Cic-L proteins are frequently separated by short (<150 pb) introns, which do not contain the first *cic-S* exon encoding the N2 repressor motif (LYLQCLL) nor its upstream promoter region (Fig. 6A). Thus, while we cannot rule out the possibility that other short isoforms of Cic exist in non-dipteran species (e.g. expressed from other alternative promoters within *cic*), a form equivalent to dipteran Cic-S (containing the N2 motif) is clearly absent in those species. Therefore, the simplest interpretation of these genomic organizations is that the Cic-S isoform and its N2 motif originated after the expansion of the above *cic-L* intron during the early radiation of dipterans; an alternative scenario, where the Cic-S isoform was already present in early insects, appears much less likely, since this would involve the independent loss of this isoform in each of the non-dipteran branches examined.

These findings indicate that Cic-L represents the ancestral isoform of Cic in insects that gave rise to Cic-S in dipterans. To further test the significance of this evolutionary change, we compared the activities of the *Drosophila* Cic-L and Cic-S isoforms in early embryogenesis. The function of Cic-L has not been studied at the molecular level, and it is even unclear whether it functions as a repressor [2]. To assay Cic-L repressor activity in the early embryo, we generated a transgene expressing Cic-L under the control of the maternal *cic-S* promoter (Fig. 6C; Materials and Methods). This construct drives efficient accumulation of Cic-L in blastoderm nuclei (Fig. 6D), but does not rescue the embryonic *cic* phenotype (Fig. 6F, H), indicating that it cannot replace Cic-S in repressing the terminal gap genes. Since Cic-L lacks the N2 motif, we then tested a Cic-L derivative carrying the N2 sequence inserted N-terminal to the HMG-box (Fig. 6C). Strikingly, this protein (Cic-L^N2^) showed significant, although not complete, rescue of the embryonic *cic* mutant phenotype (Fig. 6E, G, I). This indicates that the *Drosophila* Cic-S and Cic-L isoforms have very different molecular activities, and that evolution of the N2 motif represented a key innovation for Cic repressor function in the early embryo.

## Discussion

We have shown that Cic proteins exhibit both Gro-dependent and -independent activities, and that this functional diversity is associated with the origin of the Cic-S isoform and the N2 motif in dipterans, approximately 250 million years ago. By comparison, other functional attributes of Cic such as their sensitivity to RTK signaling and their binding to specific sites in DNA, are more broadly conserved and therefore probably more ancient. For example, the MAPK-interacting domain of *Drosophila* Cic (C2) is clearly recognizable outside the dipterans [7], and Cic is downregulated by RTK signaling in mammalian cells [15], [19]. Thus, while Cic proteins may have long served as sensors of RTK signaling, their mechanisms of repression appear to have evolved and adapted to fulfill new Cic functions in distinct transcriptional contexts. Below, we discuss the significance and implications of the newly evolved mechanism of Cic repression in fly embryogenesis.

Our results indicate that prior to the origin of dipterans, Cic was present in insects as a Cic-L-like isoform that lacked the N2 motif. Clearly, *Drosophila* Cic-L cannot function in the early embryo unless it carries the N2 motif from Cic-S (Fig. 6). This suggests that evolution of the N2 motif dramatically altered the mechanism of Cic repression by establishing a novel association with Gro. How, then, did the N2 motif appear? The comparison of different insect *cic* genes suggests that the N2 motif originated along with the Cic-S isoform, possibly through genomic rearrangements of intronic *cic-L* sequences that created a shorter *cic-S* transcript and subsequent evolution of a functional N2 motif via random drift. In this regard, it has been argued that short peptide sequences such as the WRPW and eh1 Gro-interacting motifs may be particularly easy to evolve by simple drift [29], [30].

The N2 motif is different from the WRPW and eh1 motifs, and we still do not know its precise mechanism of action. By analogy to the WRPW and eh-1 motifs, which bind the central pore of the Gro β propeller, it is possible that N2 also recognizes this region of Gro. If this is correct, the N2 motif should adopt a conformation across the pore that is insensitive to the *MB41* mutation, just like another Gro mutation, *MB31*, prevents binding of WRPW but not eh-1 to the pore [28]. Another, non-exclusive possibility is that N2 binds the Gro β propeller with the help of auxiliary proteins. Consistent with this idea, the WRPY motif of Runx proteins binds very weakly to Gro and this interaction depends on other accessory proteins in vivo [28], [31], [32].

What could be the functional and evolutionary significance of the new Gro-dependent mechanism of Cic repression? It seems logical to assume that Cic employs qualitatively different mechanisms of repression in the embryo (via Gro) than when acting in other contexts (presumably with other corepressors; see below). We suggest that the combined activities of Cic-S and Gro may have facilitated the evolution of the complex transcriptional network regulated by Torso signaling in modern fly embryos. This network comprises multiple Cic target genes, including *tll* and *hkb*, whose boundaries of expression are regulated by Torso-dependent gradients of Cic repression at the embryo poles [9], [10], [33], [34]. In this system, Gro itself appears to exert a regulatory function beyond its obvious role as a component of the repression machinery. Indeed, Gro is directly phosphorylated and functionally downregulated in response to Torso signaling [35], [36], and even modest changes in Gro protein levels significantly affect the threshold concentrations at which Cic represses *tll* and *hkb* [37]. This suggests a model where Torso signaling controls the expression of Cic target genes via coordinate activity gradients of both Cic and Gro. These overlapping gradients might serve as a fail-safe mechanism to ensure the correct spatiotemporal response of target genes, buffering against random perturbations in either gradient. Furthermore, Gro is a highly versatile corepressor capable of functioning in different contexts of recruitment [22]–[24], [38], which may explain the ability of Cic to regulate multiple targets simultaneously. For example, *tll* and *hkb* are activated by different mechanisms that are either dependent (*hkb*) or independent (*tll*) of Lilliputian, a component of the super elongation complex (SEC) [39], [40], implying that Gro is capable of counteracting both activation mechanisms. Thus, the acquisition of Gro-mediated repression by Cic may have facilitated the precise, coordinated regulation of Cic target genes in response to Torso signaling.

In contrast, Gro is mostly dispensable for other Cic functions in the wing and the follicular epithelium (Fig. 1). The Cic-S isoform is sufficient for both of these functions [4], [5], [7], raising the possibility that Cic-S acts through other corepressors in those tissues. One potential candidate is the *Drosophila* ortholog of mammalian Atxn1 (dAtxn1; [41]). In mammals, Atxn1 and the related factor Ataxin1-Like (Atxn1L; also known as Brother of ATXN1, BOAT) potentiate Cic-S repressor activity in cultured cells [14], [42], and directly interact with a short motif of Cic that is conserved in *Drosophila* Cic-S [14], [21]. dAtxn1 has been mainly studied in models of SCA1 pathogenesis [41]. Thus, future studies should examine whether dAtxn1 also mediates Cic repressor functions in development.

Finally, our results suggest that mammalian Cic proteins probably function independently of Gro, unless they have evolved other specific Gro-interacting motifs different from N2. Similarly, the mammalian Cic-S isoform must have originated independently of the dipteran Cic-S isoform, resulting in coincidental presence of Cic-S isoforms in both taxa. It will be interesting to determine whether mammalian Cic-S and Cic-L proteins also exhibit differential functional properties in their ability to regulate gene expression.

## Materials and Methods

### *Drosophila* genetics and transgenic lines

The following alleles were used: *cic^1^* [3], *cic^Q474X^* [8], *gro^E48^*, *gro^MB36^* and *gro^MB41^* [28]. *cic* mutant embryos were obtained from *cic^1^* or *cic^1^/cic^Q474X^* females, except in the experiments presented in Fig. 5D, F and H, which involved the generation of mosaic females whose germlines were homozygous for *cic^1^* using the FRT/ovoD system [43]. All *gro* embryos were derived via the FRT/ovoD system. Transgenic lines were established by P-element-mediated transformation or using the ΦC31-based integration system [44]. The *hb-h* and *hb-h^N2^* transgenes cause high levels (>98%) of female lethality and were maintained in males, either using an attached X chromosome [C(1)M3] (for X-chromosome insertions) or unbalanced (for autosomal insertions). In contrast, the *hb-h^N2-L^*, *hb-h^N2mut^*, *hb-h^C1^* and *hb-h^N1^* transgenes do not cause female lethality, even when present in two copies.

### DNA constructs

Cic-expressing transgenes were based on the original *cic* rescue construct [3], which contains the *cic-S* transcription unit flanked by its natural 5′ and 3′ regulatory sequences, and were assembled in *pCaSpeR4* or *pattB* vectors. The Cic^ΔN2^ construct lacks amino acids 3–77 of Cic-S. Cic^eh1^ contains the sequence VPLAFSISNIL instead of FQDFELGAKLYLQCLL. The Cic-L isoform used in this work is the product of cDNA *LD17181* (GenBank accession number BT100233), a fully sequenced clone identified by the Berkeley *Drosophila* Genome Project (see S2 Fig.). The LD17181 product (LD17181p) was expressed from the ATG initiator codon present in the *cic-S* rescue construct, by replacing the sequence encoding amino acids 4–19 of Cic-S with the sequence encoding amino acids 3–487 of LD17181p; note that amino acid 20 of Cic-S corresponds to amino acid 488 of LD17181p. Cic-L^N2^ was constructed by inserting an N2-containing fragment (residues 4-35 of Cic-S) at amino acid position 852 of LD17181p. All Cic derivatives have a triple HA tag (YPYDVPDYA) inserted in the same position, corresponding to amino acid 1398 of Cic-S. Hairy fusion proteins contain amino acids 1-268 of Hairy fused to the following Cic sequences: amino acids 3-35 (Hairy^N2^), and 1308-1396 (Hairy^C1^) of Cic-S, and amino acids 376-437 (Hairy^N1^) and 468-503 (Hairy^N2-L^) of LD17181p. Hairy^N2mut^, contains the sequence AYAQCLASQ instead of LYLQCLLSL.

### Embryo analyses

Embryos were fixed in 4% formaldehyde-PBS-heptane using standard procedures. In situ hybridizations were performed using digoxigenin-UTP labeled antisense RNA probes, and anti-digoxygenin antibodies conjugated to alkaline phosphatase (Roche). Immunodetection of HA-tagged Cic proteins was performed using monoclonal antibody 12CA5 (Roche) at 1∶400 dilution and secondary Alexa488-conjugated antibodies (Molecular Probes). Cuticle preparations were mounted in 1∶1 Hoyer's medium/lactic acid and cleared overnight at 60°C.

## Supporting Information

S1 Fig Expression of Hairy chimeras inactive in the *Sxl* assay. (A-D) Expression of Hairy^N2-L^, Hairy^N2mut^, Hairy^C1^ and Hairy^N1^ proteins under the control of the *hb* promoter (see Fig. 4). All proteins are readily detected by anti-HA immunostaining, indicating that their inability to repress *Sxl* is not due to inefficient accumulation in the embryo.
(TIF)

S2 Fig Structure of the *Drosophila cic* locus and two main transcripts, *cic-S* and *cic-L*, expressed from alternative promoters. White and grey boxes indicate transcribed untranslated regions and coding sequences, respectively. Sequences encoding the N1, N2, N2-L, HMG-box and C1 domains are highlighted in color. The structure of the *cic-L* transcript corresponds to the *LD17181* cDNA (see Materials and Methods). The sequence of the first exon and its immediate upstream region is shown below to indicate the positions of the annotated transcription initiation site (TIS, bent arrow) and the 5′ end of *LD17181* (arrowhead). The position of the TIS is based on RNA-seq profiles generated by the modENCODE project [47]. The translated peptide sequence is also shown in bold, with residues encoded by *LD17181* highlighted in red; thus, the LD17181p product is 5 amino acid shorter than the corresponding predicted Cic-L protein (1871 vs. 1876 residues, respectively). Genomic sequences from *Drosophila erecta* (*De*) and *Drosophila yakuba* (*Dy*) are aligned below the *melanogaster* (*Dm*) sequence; note that both species contain in-frame stop codons (asterisks) immediately upstream of the N-terminal methionine, supporting the predicted initiation of translation.
(TIF)
