# Supplementary figures and images for: Origins of Context-Dependent Gene Repression by Capicua
Source: PLoS Genet. 2015 Jan 8;11(1):e1004902. doi: 10.1371/journal.pgen.1004902 (PMC4287436; doi:10.1371/journal.pgen.1004902)

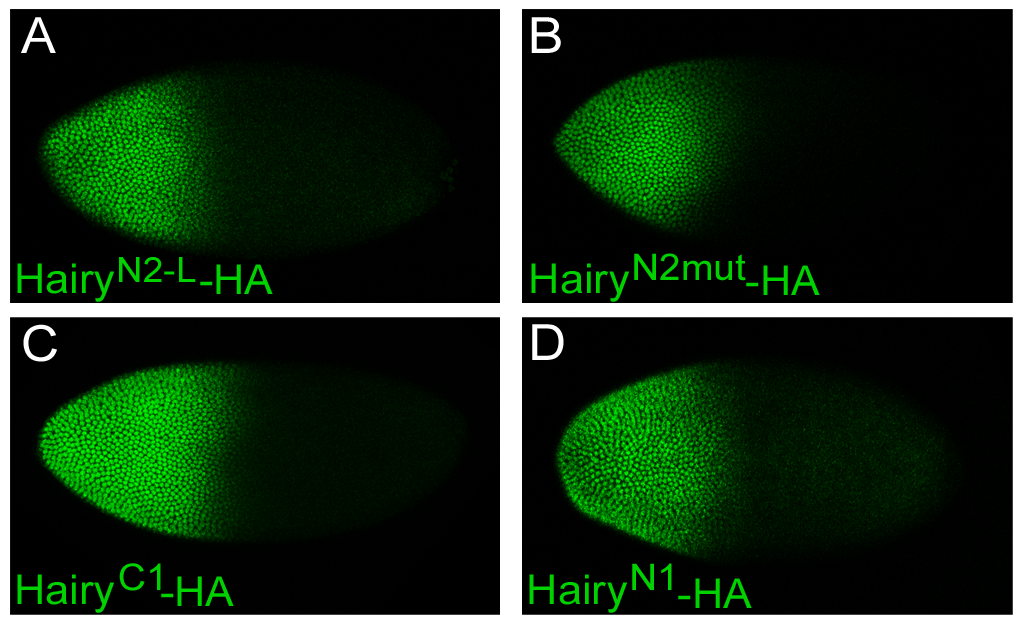

Supplement: S1 Fig — Expression of Hairy chimeras inactive in the Sxl assay. (A-D) Expression of HairyN2-L, HairyN2mut, HairyC1 and HairyN1 proteins under the control of the hb promoter (see Fig. 4). All proteins are readily detected by anti-HA immunostaining, indicating that their inability to repress Sxl is not due to inefficient accumulation in the embryo. (TIF) [file pgen.1004902.s001.tif]

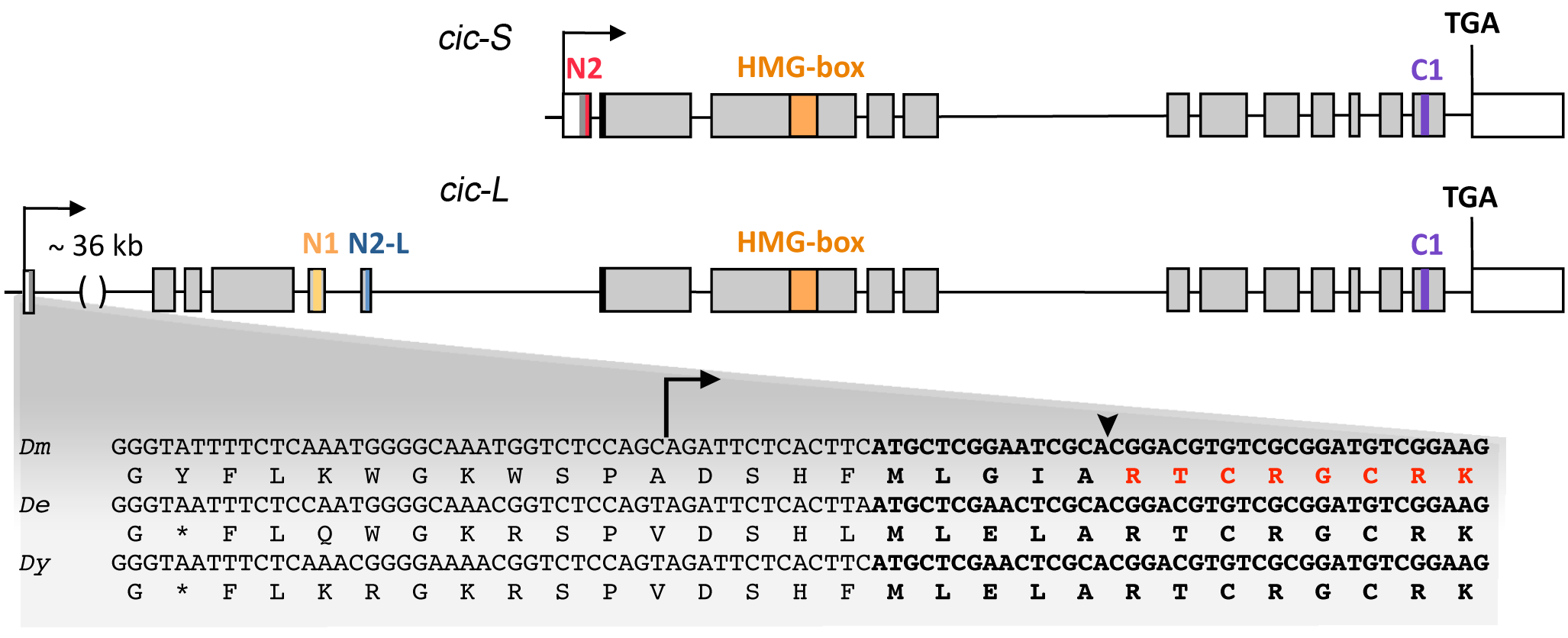

Supplement: S2 Fig — Structure of the Drosophila cic locus and two main transcripts, cic-S and cic-L, expressed from alternative promoters. White and grey boxes indicate transcribed untranslated regions and coding sequences, respectively. Sequences encoding the N1, N2, N2-L, HMG-box and C1 domains are highlighted in color. The structure of the cic-L transcript corresponds to the LD17181 cDNA (see Materials and Methods). The sequence of the first exon and its immediate upstream region is shown below to indicate the positions of the annotated transcription initiation site (TIS, bent arrow) and the 5′ end of LD17181 (arrowhead). The position of the TIS is based on RNA-seq profiles generated by the modENCODE project [47]. The translated peptide sequence is also shown in bold, with residues encoded by LD17181 highlighted in red; thus, the LD17181p product is 5 amino acid shorter than the corresponding predicted Cic-L protein (1871 vs. 1876 residues, respectively). Genomic sequences from Drosophila erecta (De) and Drosophila yakuba (Dy) are aligned below the melanogaster (Dm) sequence; note that both species contain in-frame stop codons (asterisks) immediately upstream of the N-terminal methionine, supporting the predicted initiation of translation. (TIF) [file pgen.1004902.s002.tif]
